# RNAmap2D – calculation, visualization and analysis of contact and distance maps for RNA and protein-RNA complex structures

**DOI:** 10.1186/1471-2105-13-333

**Published:** 2012-12-21

**Authors:** Michal J Pietal, Natalia Szostak, Kristian M Rother, Janusz M Bujnicki

**Affiliations:** 1 Laboratory of Bioinformatics and Protein Engineering, International Institute of Molecular and Cell Biology in Warsaw, ul. Ks. Trojdena 4, PL 02-109, Warsaw, Poland; 2 Laboratory of Bioinformatics and Protein Engineering, International Institute of Molecular and Cell Biology in Warsaw, ul. Ks. Trojdena 4, Warsaw, PL 61-614, Poland

**Keywords:** Contact maps, Distance maps, RNA secondary structure, RNA base pairing, RNA stacking, Protein-RNA complex, Docking

## Abstract

**Background:**

The structures of biological macromolecules provide a framework for studying their biological functions. Three-dimensional structures of proteins, nucleic acids, or their complexes, are difficult to visualize in detail on flat surfaces, and algorithms for their spatial superposition and comparison are computationally costly. Molecular structures, however, can be represented as 2D maps of interactions between the individual residues, which are easier to visualize and compare, and which can be reconverted to 3D structures with reasonable precision. There are many visualization tools for maps of protein structures, but few for nucleic acids.

**Results:**

We developed RNAmap2D, a platform-independent software tool for calculation, visualization and analysis of contact and distance maps for nucleic acid molecules and their complexes with proteins or ligands. The program addresses the problem of paucity of bioinformatics tools dedicated to analyzing RNA 2D maps, given the growing number of experimentally solved RNA structures in the Protein Data Bank (PDB) repository, as well as the growing number of tools for RNA 2D and 3D structure prediction. RNAmap2D allows for calculation and analysis of contacts and distances between various classes of atoms in nucleic acid, protein, and small ligand molecules. It also discriminates between different types of base pairing and stacking.

**Conclusions:**

RNAmap2D is an easy to use method to visualize, analyze and compare structures of nucleic acid molecules and their complexes with other molecules, such as proteins or ligands and metal ions. Its special features make it a very useful tool for analysis of tertiary structures of RNAs. RNAmap2D for Windows/Linux/MacOSX is freely available for academic users at http://iimcb.genesilico.pl/rnamap2d.html

## Background

RNAs and proteins are linear polymers composed of a limited set of building blocks (ribonucleotide and amino acid residues, respectively) that may spontaneously fold into complex three-dimensional shapes [1,2]. In both RNA and proteins, the order of building blocks held together by covalent bonds is called the primary structure, the local conformation of the chain stabilized mostly by hydrogen bonds is the secondary structure, while the path of the chain in three dimensions (3D) resulting from various long-range interactions is the tertiary structure. It is known that many proteins and RNAs undergo conformational transitions. Macromolecules and their parts may also exhibit structural disorder, i.e. fluctuations between many different conformations [3,4]. The functions of proteins and RNAs typically involve physical interactions with other molecules in the cell, which are dependent on the structure and plasticity of the interacting partners. Thus, the functions of proteins and RNAs alike depend on the 3D structure and dynamics of these molecules, which in turn are encoded in their linear, i.e. unidimensional (1D) sequences [5].

While three-dimensional macromolecular structures represent an information-rich framework for studying biological functions, visualizing and analyzing them is difficult both for humans and computer programs. Two-dimensional (2D) flat images are more readily discernible to the eye and more memorable than complex 3D images. As a matter of fact, 3D structures of macromolecules (including proteins, nucleic acids, and their complexes), can be represented as square symmetrical matrices containing data about the proximity of residue or atom pairs in the molecule [6]. Values stored in the matrix may represent e.g. euclidean distances between particular atoms, such matrix is then called a distance map). If only binary information about residue-residue interactions is included, a matrix is called a contact map. In a contact map, a residue-residue interaction may be qualified by an euclidean distance below a given threshold or by a particular type of contact. The representation of macromolecular structure by its 2D map is independent on the coordinate frame (i.e. makes the representation invariant to rotations and translations), which makes it useful for both visualization and structure comparison. Contact maps can be also enriched with additional information, e.g. to discriminate different types of contacts or to indicate the chirality in 3D (handedness), which is otherwise lost upon the conversion to a 2D representation.

It has been demonstrated that the 3D structure of a protein (and, by analogy, any linear polymer that forms a compact structure) can be recovered from its contact map representation, with the reconstructed and original structures similar up to the resolution of the contact map representation [7]. This rationalizes the development of computational tools for analysis and visualization of macromolecular 2D maps, as an alternative to dealing directly with 3D structures. In fact, complex structures of RNA molecules have been traditionally described as 2D diagrams, exemplified by the popular “cloverleaf” representation of the tRNA molecule. Such diagrams emphasize “orthodox” pairing of bases with their Watson-Crick edges, and with the use of additional symbols or colors they can also display other types of pairing, using the Hoogsteen and sugar edges [8]. However, a single nucleotide residue can interact with more than one nucleotide at a time (stacking using two faces, base-pairing using three edges, and interacting with the phosphate group). In this situation secondary structure plots become illegible due to crossing of multiple lines indicating long-distance contacts. On the other hand, the same type of information can be represented (and visualized) with a square symmetrical matrix containing data about different types of interactions e.g. discriminated by the use of different colors. With only a little training, one can learn to quickly distinguish e.g. helices and complex tertiary arrangements, such as pseudoknots. Similarities between distantly related RNAs can be seen easily in contact maps, even when the 3D structures superimpose poorly.

The development of computational tools for molecular structure visualization must keep up with the growing amount of bioinformatics data and the definition of new data types. Structure visualization, analysis, and annotation tools play an increasingly important role in research, contributing significantly to understanding the biological function of macromolecules. There is a number of tools capable of calculating, displaying, and analyzing protein contact maps, including VMD [9], SeqX [10], PConPy [11], or CMView [12]. While some of these methods are able to display contacts of the protein component to the nucleic acid ligand, they do not produce a 2D map if RNA structure alone is provided as input, and they do not discriminate between different types of nucleotide interactions. As more and more RNA structures are determined by experimental methods or predicted by computational techniques, it becomes increasingly important to reinforce current state-of-art 3D RNA modeling tools with 2D visualization capabilities. Thus, we developed a computer program dedicated to structural maps of RNAs and RNA-protein or ligand-complexes, with particular emphasis on visualization of different types of interactions mediated by edges or faces of nucleotide bases, i.e. pairing and stacking.

## Implementation

### Programming language, systems, external programs

RNAmap2D was developed based on its predecessor PROTmap2D [13]. The core programming language of RNAmap2D is Python. The program uses the Biopython [14] and PyCogent [15] libraries to handle PDB structures.

RNAmap2D is available for Linux, Windows and MacOSX. To create a Windows executable version of the program, we used the py2exe 0.6.9 tool and to build a MacOSX version, py2app 0.3.6, respectively. Hardware requirements for RNAmap2D are very modest as of 2012, i.e. 1 GHz processor and 512 MB RAM memory.

In analogy to a number of programs for determination of protein secondary structure from 3D coordinates [16] that differ from each other in definition of structures and algorithms for their detection, there exist various tools for determination of contacts from RNA 3D structures. We have adapted RNAmap2D to use RNAView [17] for base pair calculation. However, RNAView is not available for Windows, therefore we added our own procedure for calculating base/nucleotide pairs, developed in ModeRNA [18]. For every pair of residues considered, it superimposes reference frames of all known pairs types as well as predicts the presence of H-bonds characteristic for a given pair type based on interatomic distances and angles. This makes the latter procedure sensitive to distortions of the relative conformation of the nucleotide. According to our tests this procedure agrees with RNAView in assignment of ~95% of canonical base pairs. At startup, RNAmap2D checks for the presence of the installed RNAView program, and uses it by default for base pair interactions classification, while in its absence our modules are used.

### User skills

RNAmap2D requires no programming or scripting skills to make use of all of its features, regardless of the platform. The Windows and MacOSX versions require no installation, except for downloading and unpacking the distribution file. Linux distribution is a set of bytecode Python files that require the installation of all modules cited at the beginning of this section prior to the use of the program. Users are provided with a comprehensive manual and a readme file that explains all installation steps. All versions provide the same functionalities except for the above-mentioned absence of RNAView for the Windows platform.

Our program has an easy-to-use graphic user interface that allows for customizing the final contact or distance map, and by setting all the important parameters. RNAmap2D navigation scheme design allows for making a step back from the final map view to the main options panel in order to refine parameters and promptly visualize the desired output. The intermediate results of calculations are stored in the computer memory to keep the data processing time at a minimum.

Distance and contact maps can be saved and uploaded as text files in a variety of formats, including e.g. the ones used in CASP or by the PHYLIP package. Maps can be also exported and uploaded as Microsoft Office Excel™ spreadsheets. Finally, images of maps can be exported as PNG, BMP, and TIFF files.

### Speed

To optimize the speed of RNAmap2D, we used C and C++ libraries that are accessible under Python: wxPython (for the graphical user interface), Numeric and NumPy (for most numeric calculations). Also, the core calculation routine in RNAmap2D is the determination of distances and contacts between atoms and residues. We used the KDTree algorithm [19] as implemented in Biopython, which has log-linear calculation time, fastest known to date. As an example, for a molecule of the size of 100 nucleotide residues, the calculation time reaches 1000 time units (typically below a second for obtaining a raw list of contacts from a 3D structure). It compares favorably e.g. with an alternative approach used by the PConPy program for protein contact maps [11] that relies on a naïve double loop for calculating a contact map. For the molecule of identical size, the calculation time would reach 4950 units, which is five times slower. This is because time complexity of most such algorithms is of the order of O(n^2^). Please note however, that the calculation time for RNA molecules longer than 200 bases can be quite long if no RNAView plugin is installed. Bearing this in mind, we designed an additional “no pairings (fast)” contact map calculation mode that bypasses the pairing calculation algorithm and can be used alternatively for large RNA structures, in order to obtain a raw contact map very quickly.

## Results and discussion

Based on our previous experience with protein 2D map analysis and core parts of the PROTmap2D code [13], we developed RNAmap2D, a standalone tool for calculation, visualization and analysis of contact and distance maps of nucleic acid structures and structures of protein-nucleic acid complexes. PROTmap2D was designed to perform various tasks on proteins only. As a consequence, it has no generic or specific capabilities that could address tasks specific to nucleic acids or complexes formed by nucleic acids. Also, PROTmap2D cannot visualize contacts made by macromolecules with ligands or ions. On the other hand, the interaction of RNA with ions is essential for the formation and stability of its 3D structure [20], therefore RNA-ion contacts shouldn’t be neglected in structural analyses. RNAmap2D is therefore an independent program, with multiple features specific for RNA, and not simply an upgrade of PROTmap2D. Our method can serve to analyze DNA as well as RNA. However we expect that because of regularity of DNA structures, this type of nucleic acid will not be frequently analyzed on the 2D level, hence the program’s name includes only RNA as the key input molecule. For simplicity, in this article we refer mostly to RNA alone.

RNAmap2D can calculate a contact map or a distance map of an RNA structure, compare two alternative 3D models of RNA (e.g. predicted structure versus experimentally solved one). Our program can analyze an ensemble of structures (e.g. the content of a PDB file solved by the NMR method), with two alternative statistics measures. RNAmap2D is also capable of visualizing a contact map of protein-RNA complex, and a series of such models, e.g. originating from a protein-RNA docking experiment. RNAmap2D can also calculate, visualize and export RNA secondary structure in a common dot-bracket format. Secondary structure can be also imported and displayed as a 2D diagram – in this case all contacts visualized by RNAmap2D will correspond to Watson-Crick base pairs, as other types of contacts are not represented in traditional secondary structure representations.

RNAmap2D generates interactive 2D maps, with a possibility to zoom in onto particular fragments. Zooming is enabled when a user presses a mouse button over a map and drags a rectangle-shaped area. The content of this area is shown in a separate window, with a sequence ruler, range indicator and a possibility of resizing. Also, when a user hovers a mouse cursor over a specific contact, a window appears with information about the residue pair, including residue index, residue name and binary (Y/N) contact information. Additional interaction schemes specific to different tasks are available, such as color panels, fields to input certain value limits etc., which are described below in specific sections.

Output options for 2D maps include a number of image formats (BMP, GIF, JPG, PNG, TIFF), and various text formats developed initially for storing the results of comparative analyses of protein sequences and structures: CASP [21], EVA [22], PHYLIP [23] and CLANS [24]. RNAmap2D can also encode maps in formats such as CSV or MS Excel™ spreadsheets, which can be easily read and analyzed with many third-party tools. The program is also capable of reading a map encoded as a text or Excel file, e.g. a file exported from RNAmap2D and edited in a third-party program.

**Figure 1:**
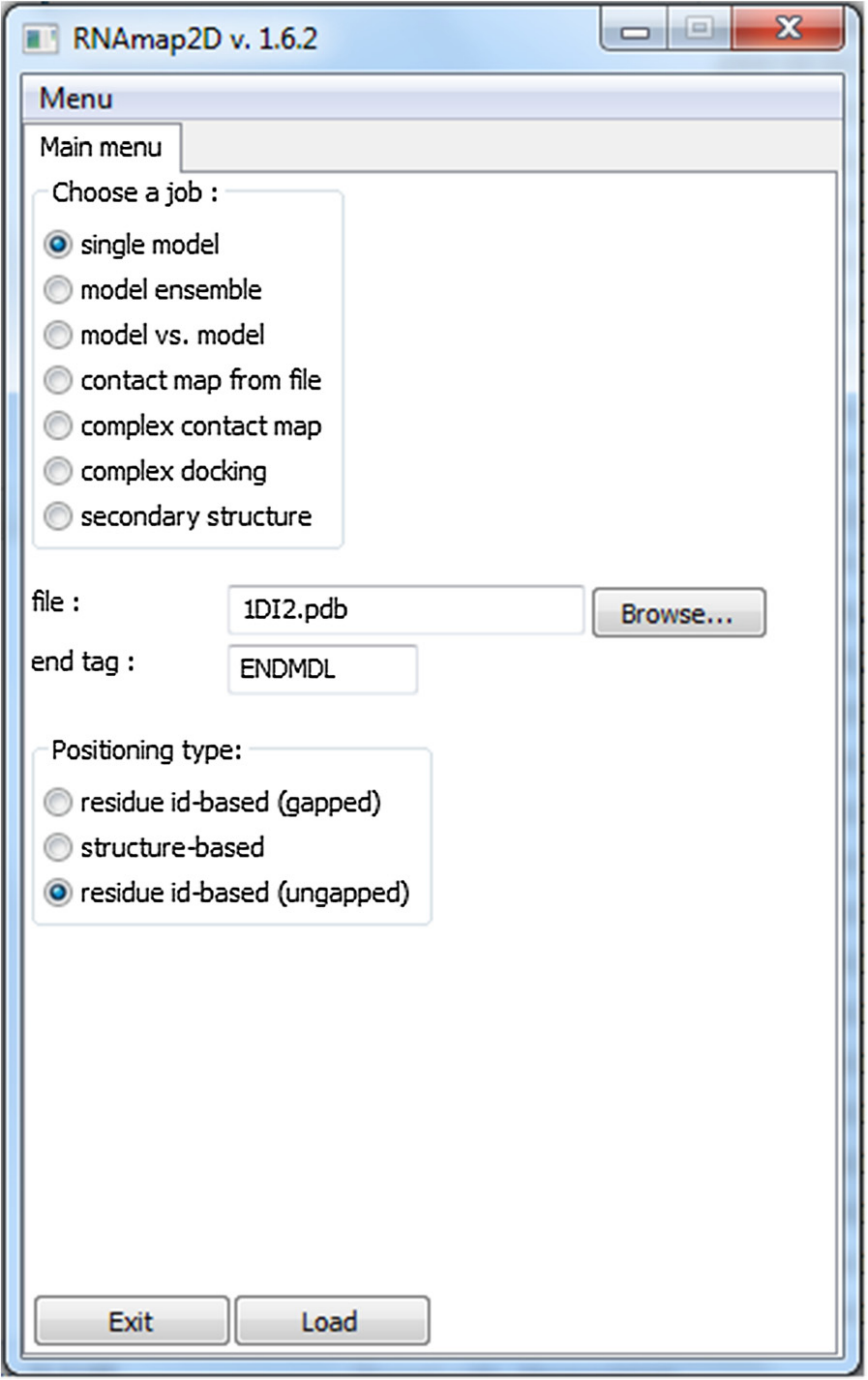
**RNAmap2D user interface. **The main menu screen of RNAmap2D, which serves as the entry panel for all analyses. The user is asked to select the type of analysis (“a job”), provide the input filename, and indicate whether the program should use residue numbers from the input file or recalculate them. Additionally, for multi-model files (e.g. model ensemble analysis), the user is asked to define the tag that separates individual models in the input file.

In the section below we highlight some of the tasks of RNAmap2D that we find most typical in our own research on RNA structures. All tasks and options are extensively documented in the User’s Manual, which is provided on the program’s website. The program is also accompanied by a Tutorial, which intends to demonstrate all standard features for a user without any prior knowledge. A set of PDB structures and other files is also provided, so that users do not have to search for appropriate input in order to test and see all the features described in the Tutorial (Figure 1).

### RNA contact map

RNAmap2D works on the level of residues rather than individual atoms. A user can specify the definition of atoms used to calculate the presence of a contact: the type of atoms to be included in calculations (the options per residue are: single atoms - C1^′^, C4^′^, O5^′^, N1/N9 for purines and pyrimidines respectively, or multiple atoms - all or heavy/non-hydrogen), a maximal distance (in Å) between the specified atoms to form a contact, and a minimal residue separation along the sequence (e.g. to exclude contacts between consecutive residues that are connected by covalent bonds). If multiple atoms are considered, it is sufficient for any pair of atoms from two residues to fall below the distance threshold to have this residues classified as in contact; RNAmap2D does not generate a map of contacts between individual atoms, only maps information about proximal atoms on the respective residues. However individual heteroatom records found in PDB files that represent ions or ligand atoms are treated as separate residues. If the PDB file contains several models, the user can choose a specific model to be analyzed by the program. For oligomeric structures, in most analyses it is also possible to specify chain identifiers to limit the analysis to certain chains of the molecule. Auxiliary options are: graphic sequence delimiter (ruler) and grid-like lines that separate chains (chain borders).

**Figure 2:**
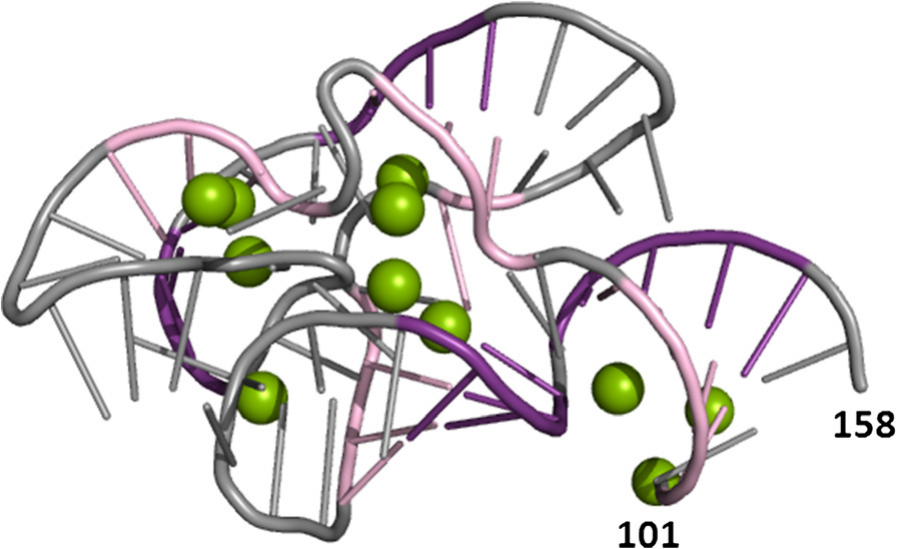
**A traditional 2D rendering of the 3D structure for the 23S rRNA fragment (PDB ID: 1HC8, chain C). 5’ and 3’-terminal residues are numbered. **Nucleotide residues involved in Watson-Crick base-pairing are shown in pink and violet. Mg^2+ ^ions are shown as green balls. The limitation of this representation is that a single atom can have only one color and the use of multiple colors to illustrate different types of interactions can become overwhelming.

**Figure 3:**
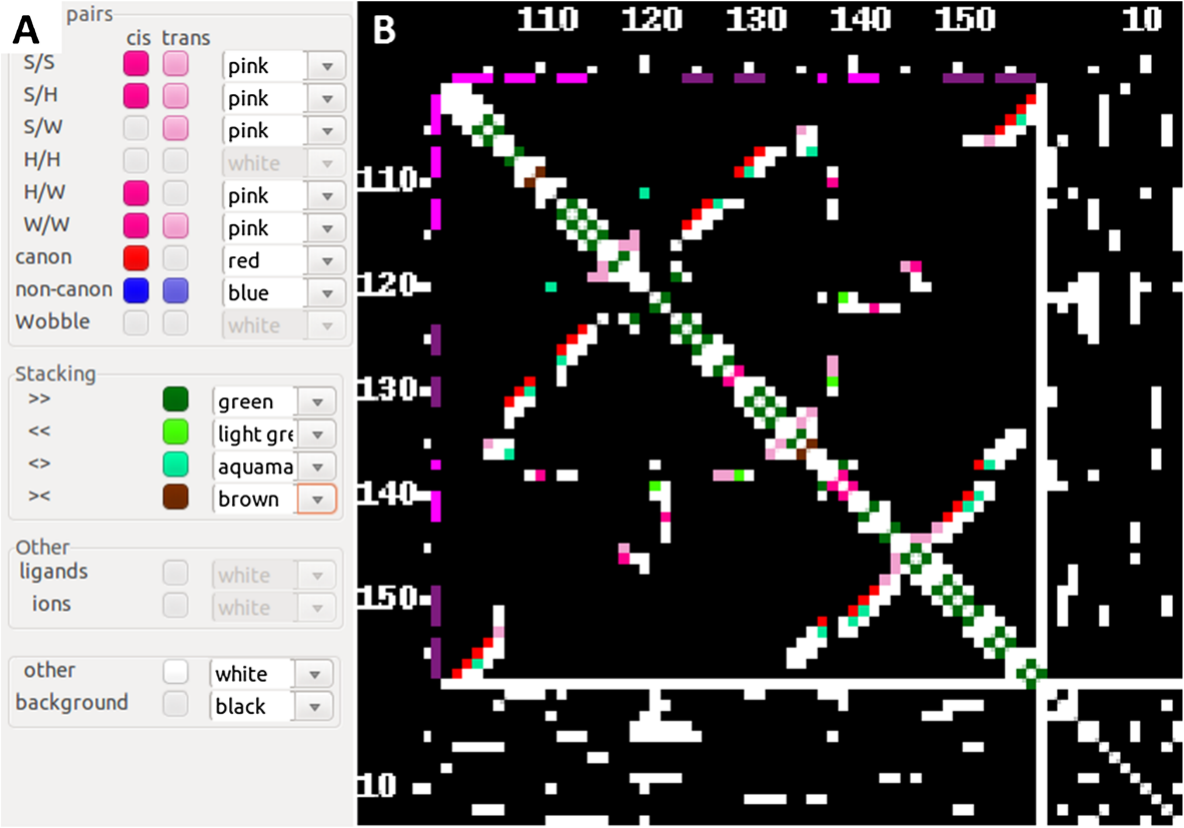
**Coloring capabilities of RNAmap2D. A) **The coloring panel of RNAmap2D, with a selection of several types of pairing, stacking, and other interactions. Note that all types of pairings and stacking are identified by the program before the panel is displayed, and options corresponding to interactions undetected in the input file are disabled; **B) **A contact map of 23S rRNA fragment (PDB ID 1HC8_A, 3D structure as shown in Figure 2), calculated for N1/N9 atoms using the threshold of 9.5 Å and colored according to the code in panel A. The top left square represents the RNA chain, while the bottom right square shows Mg^2+ ^ions and other heteroatoms classified as either ions or ligands. The symmetrically oriented upper right and bottom left sections indicate contacts between nucleotide residues and ions. Purple and violet bars along the sequence ruler indicate RNA secondary structure.

A contact map can be visualized as a black-and-white picture, with no distinction between contacts, symbolized as white dots against black background (no contact). Following the calculation of the contact map, the user can choose independent color schemes in order to highlight and visualize contacts that belong to one of the 12 base pair families, three special groups (canonical, non-canonical and Wobble), or to one of the four stacking classes [8]. The coloring is indicated by invoking an option panel, which appears as a separate window. RNAmap2D presents an option to color contacts made by ligands or ions, which appear as additional “residues” following the sequence of macromolecules (Figures 2 and 3).

### RNA distance map

**Figure 4:**
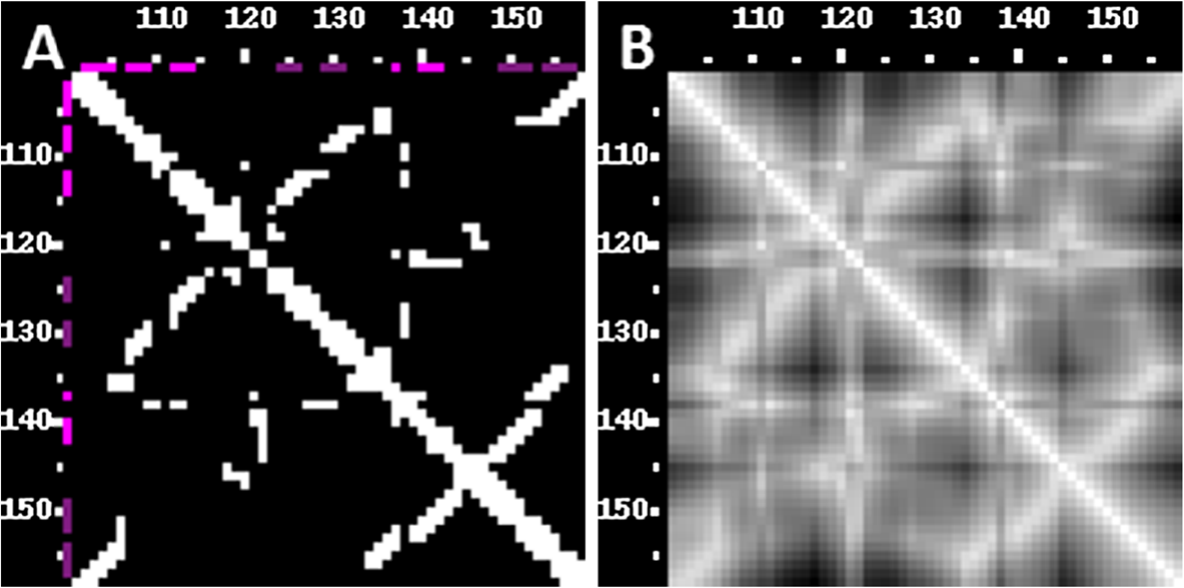
**Contact vs distance maps. A) **A contact map of 23S rRNA fragment (the same structure as in Figure 2) calculated for N1/N9 atoms with a 9.5 Å distance threshold. All contacts are shown as white squares. Purple and violet bars along the sequence ruler indicate RNA secondary structure. **B) **A distance map for the same molecule and metric. The contact map shows only binary information, while the distance map represents the degree of proximity by the shades of grey (white color symbolizes a distance equal to zero).

Distance maps are richer in data than contact maps, and essentially preserve all the information required to infer the details of 3D structure, with the exception of the handedness, which can be imposed on a higher level of reasoning, because we know the stereochemistry of nucleic acid structures (e.g. A-RNA helix must be right-handed). As with contact maps, RNAmap2D does not generate a map of distances between individual atoms; if multiple atoms are considered per residue, the shortest distance is taken for each pair of residues. One option for visualization of distance maps is the contact map view. This feature allows the user to optionally convert a distance map into a series of contact maps, calculated at different thresholds. New contact maps are calculated and visualized instantly, as the user changes the maximal distance parameter defining a contact (cutoff), by using a mouse roller. This enables a user to establish a subjectively optimal distance threshold parameter value for the purpose of obtaining a contact map that highlights certain molecular features (e.g. the minimal spatial proximity of two nucleotide residues to form a relevant interaction). Following the calculation of a distance map, the user is given an option to define the absolute distance limits, by entering minimal or maximal distance and the minimal sequential separation of nucleotides to be considered. The results are visualized instantly as well (Figure 4).

### RNA secondary structure

**Figure 5:**
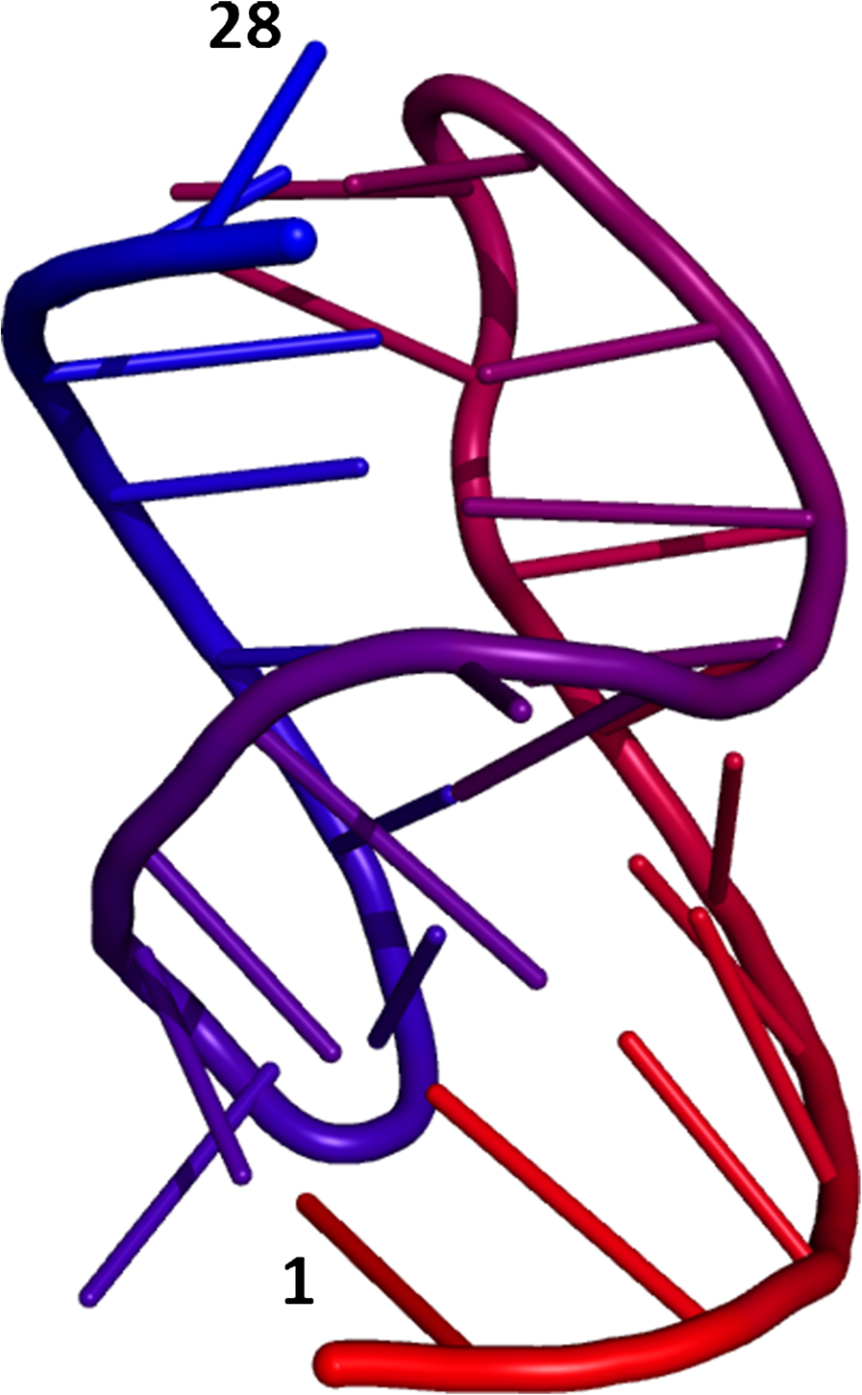
**An example RNA structure with a pseudoknot. **A projection of a 3D structure of a viral RNA pseudoknot (crystal structure, PDB id 1L2X) in the simplified cartoon format (backbone as a ribbon, nucleotide residues as sticks). 5’ and 3’-terminal residues are labeled. The structure is colored as a rainbow spectrum, from 5’ (red) to 3’ (blue) termini.

**Figure 6:**
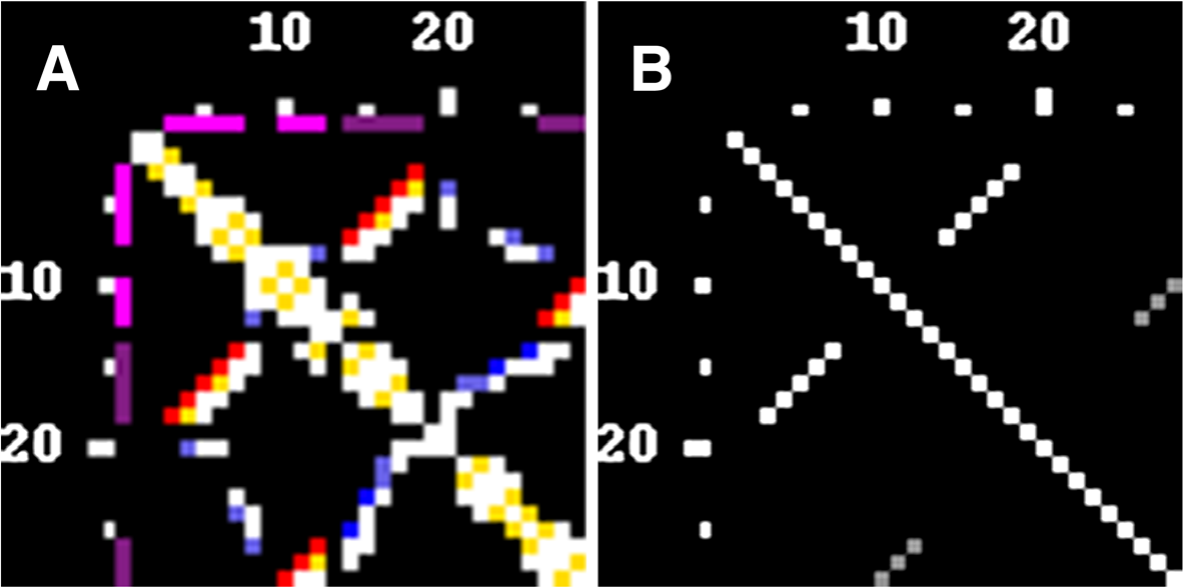
**RNA secondary structure. **Base pairs have been classified by RNAView. **A) **A contact map (PDB id 1L2X, see: Figure 5 for a traditional rendering) calculated using the threshold of 9.5 Å, using N1/N9 atoms; canonical base pairs are shown in red, other base pairs are in blue, stacking interactions are shown in yellow and other contacts are in white. **B) **The RNA secondary structure (Watson-Crick base pairs only) was saved in the Vienna format and reloaded in the contact map form. The grey color indicates residues participating in pseudoknot pairings.

The calculated secondary structure can be written in the popular Vienna [25] format as a dot-bracket string. RNAmap2D recognizes pseudoknots, for which special bracket symbols (“[“ and “]” characters plus others if needed) are used in the dot-bracket string. If an RNA secondary structure file is uploaded, RNAmap2D can display the Watson-Crick base pairs as a simplified contact map. RNAmap2D can read the Vienna, CT, and BPSeq secondary structure formats. Pseudoknotted base pairs are shown in grey (Figure 5 and 6).

### Contact maps of complexes involving different types of molecules

**Figure 7:**
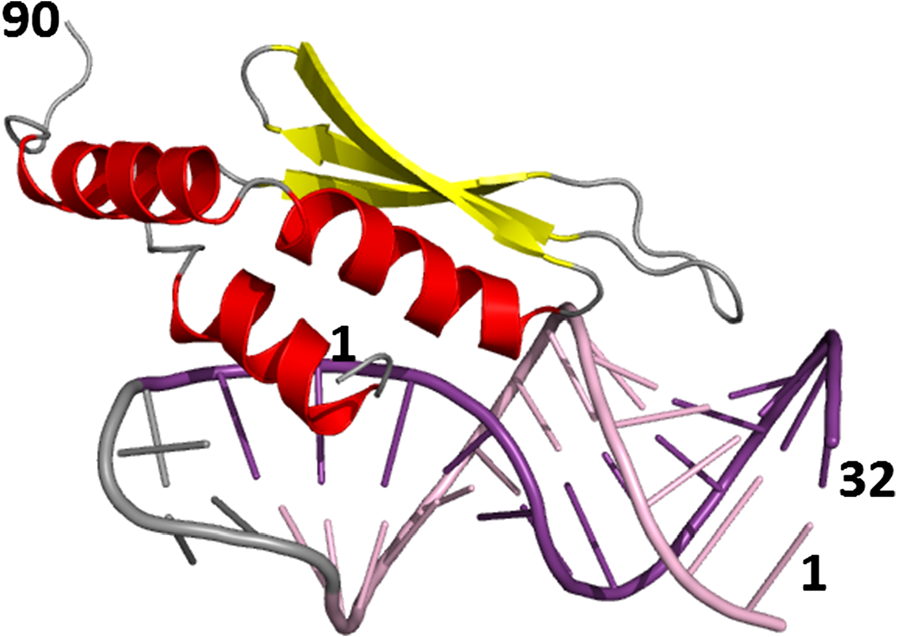
**Protein-nucleic acid complex. **A double-stranded RNA binding domain of *S*. *cerevisiae *RNAse III in complex with an AAGU tetraloop hairpin (PDB code: 2LBS, only the first model of the NMR ensemble is shown). The protein chain is represented as symbolic secondary structure cartoons: helices in red, strands in yellow. RNA molecule is represented as bonding sticks and colored according to secondary structure (base-paired residues are shown in violet and pink). Terminal residues are labeled.

**Figure 8:**
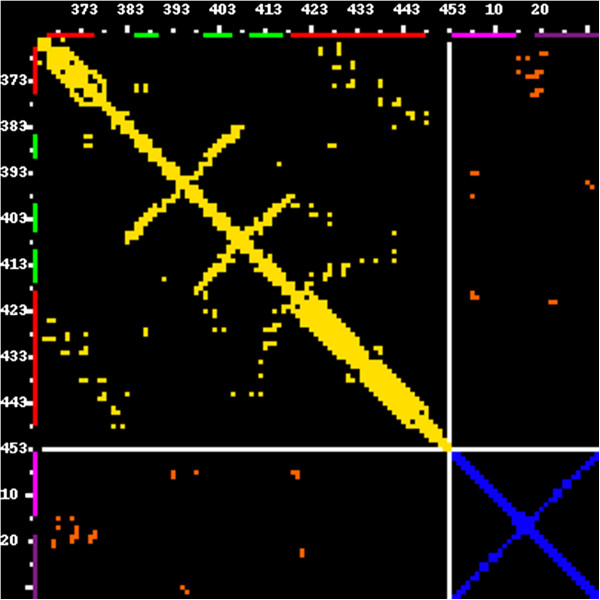
**Contact map of a protein-nucleic acid complex. **A double-stranded RNA binding domain of *S*. *cerevisiae *RNAse III in complex with an AAGU tetraloop hairpin (PDB code: 2LBS, only the first model is shown, the same as in a traditional rendering in Figure 7). In a contact map picture, protein contacts are displayed in yellow, RNA contacts are blue, and the protein-RNA interface contacts are displayed in orange. Metrics preset: all-atoms for RNA and protein molecules, 3.5 Å contact threshold. However, the atom type and the distance threshold can be set independently for RNA-RNA, protein-protein, and protein-RNA contacts. In the protein part of the map, red and green bars indicate secondary structures (alpha helices and beta strands, respectively), read directly from the PDB file. In the RNA part of the map, purple and violet bars indicate RNA secondary structure computed using the RNAView program.

Nucleic acids usually function in complex with proteins, and many structures of protein-nucleic acid complexes have been determined experimentally. In the PDB database, there are 3575 such entries as of April 11th 2012, contributing to 4.4% of the total PDB records. RNAmap2D is capable of either extracting nucleic acid chains from a complex structure in order to visualize them separately or it can include the coordinates of the protein component and display protein-nucleic acid interactions. A contact map of a protein-nucleic acid complex is visually divided into three distinct parts for intra-protein contacts, intra-nucleic acid contacts and intermolecular contacts between the two entities. These three categories can be colored differently for better visual distinction. Whenever possible, RNAmap2D utilizes protein secondary structure definitions found in a PDB file, and displays them (red for helices, green for strands) along the ruler of the contact map, alongside the RNA secondary structure bars (Figure 7 and 8).

### Comparison of contact maps

RNAmap2D can compare two RNA structures in two modes: molecules that have an identical number of residues, or molecules that contain a common chain with residues with corresponding numbers. The input can be either a single PDB file with two models, or two separate files, e.g. a 3D model and a reference structure. The sequences do not need to be identical. Thus, it is possible to compare two models of the same RNA (even if they don’t cover the same range of residues, as long as there is some overlap), sub-structures of different molecules, e.g. homologs with similar structure but different sequence, etc.

**Figure 9:**
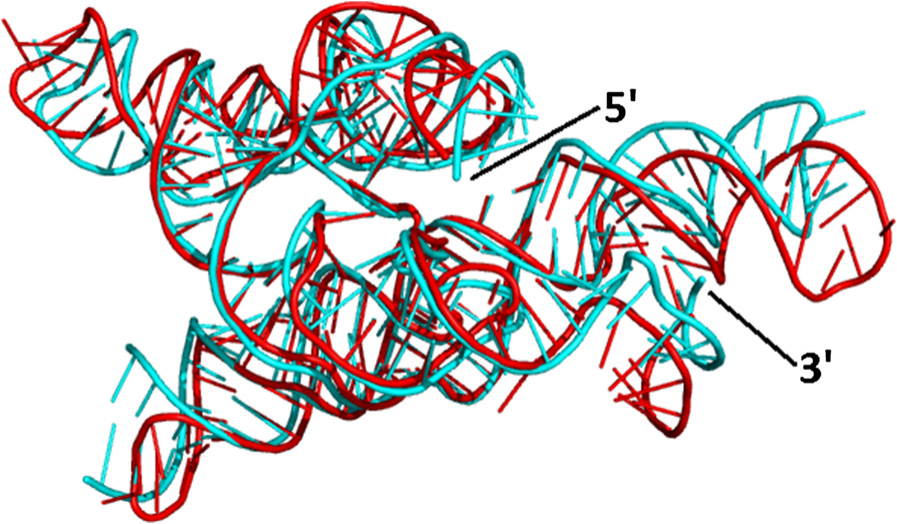
**Comparison of a group I intron crystal structure and a comparative 3D model built with the ModeRNA program, in 3D. **The homology model is compared to the crystal structure (1U6B_B), which was transformed by the deletion of 14 nt fragment to match the target sequence without major gaps. The picture shows both 3D structures aligned: crystal structure (cyan) and the model (red).

**Figure 10:**
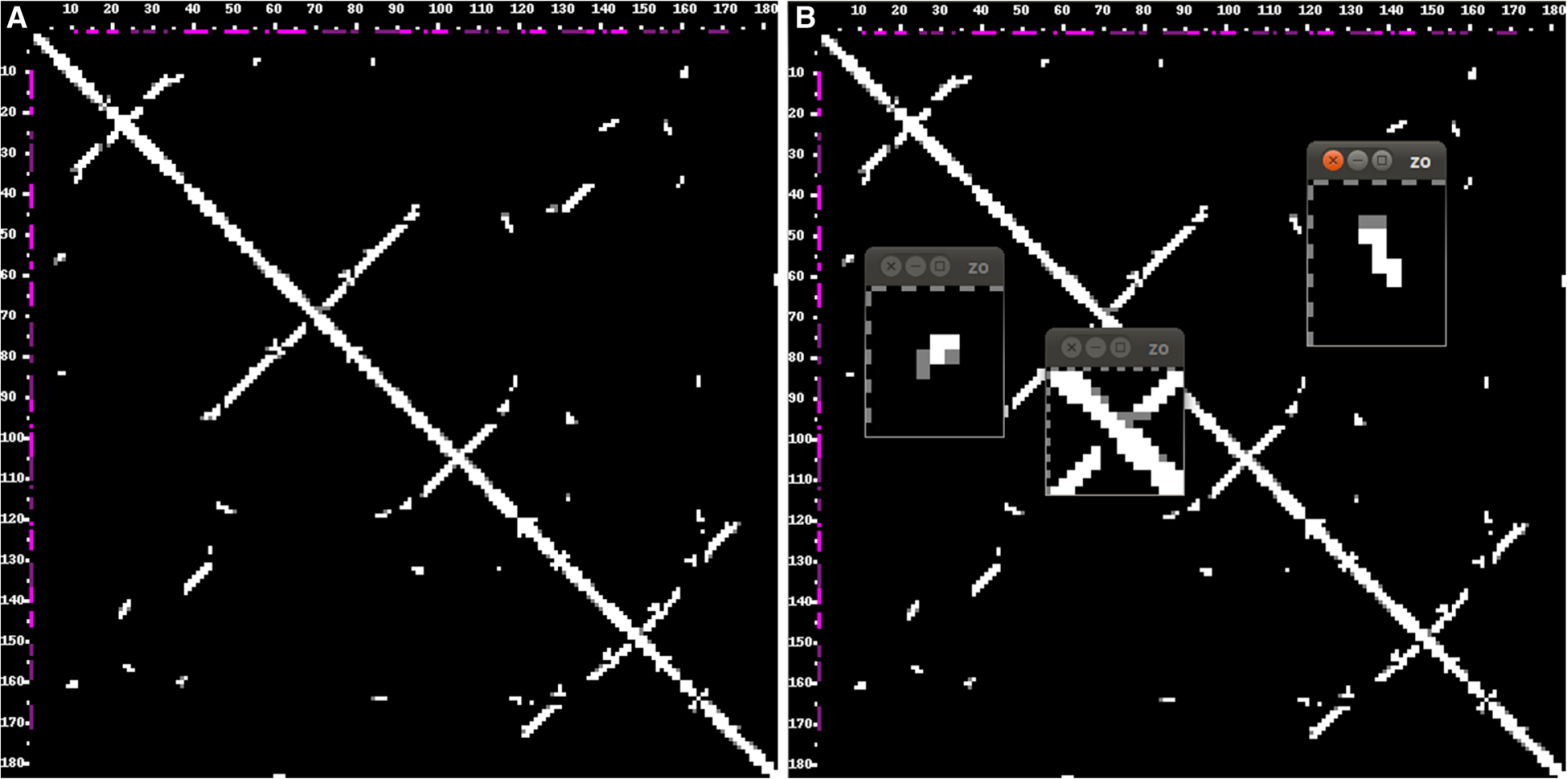
**Comparison of a crystal structure and a comparative 3D model, in 2D. **The homology model is compared to the crystal structure. Contacts have been calculated using the N1/N9 metrics and 9.5 Å threshold (1U6B_B, 3D picture shown in Figure 9). **A) **The lower left triangle displays the contacts in the crystal structure, the upper right triangle those in the model. **B) **The right picture additionally highlights three regions where the contacts differ, in particular one tertiary interaction site (left), one loop (center) and one junction (right).

In either case, two contact maps are shown concurrently, with the first structure in the lower-left triangle, the second in the upper-right triangle. Contacts common to both structures are shown as white dots, while contacts specific to either structure are in grey. In Figure 9 and 10, we compared the crystal structure of the *Azoarcus* group I intron (PDB-id 1U6B, chain B) with a 3D model for built on a template from the *Twort* phage using ModeRNA [25]. The reference structure is 198nt long and contains several tertiary contacts that were constructed using a second template from *Tetrahymena*. Twelve residues at the 3^′^ end are not present in the model. The all-atom RMSD of the model versus crystal structure is 4.3 Å. The contact map analysis allowed us to identify small differences in contacts between the model and the reference structure, mainly in loops and in the tertiary motifs.

**Figure 11:**
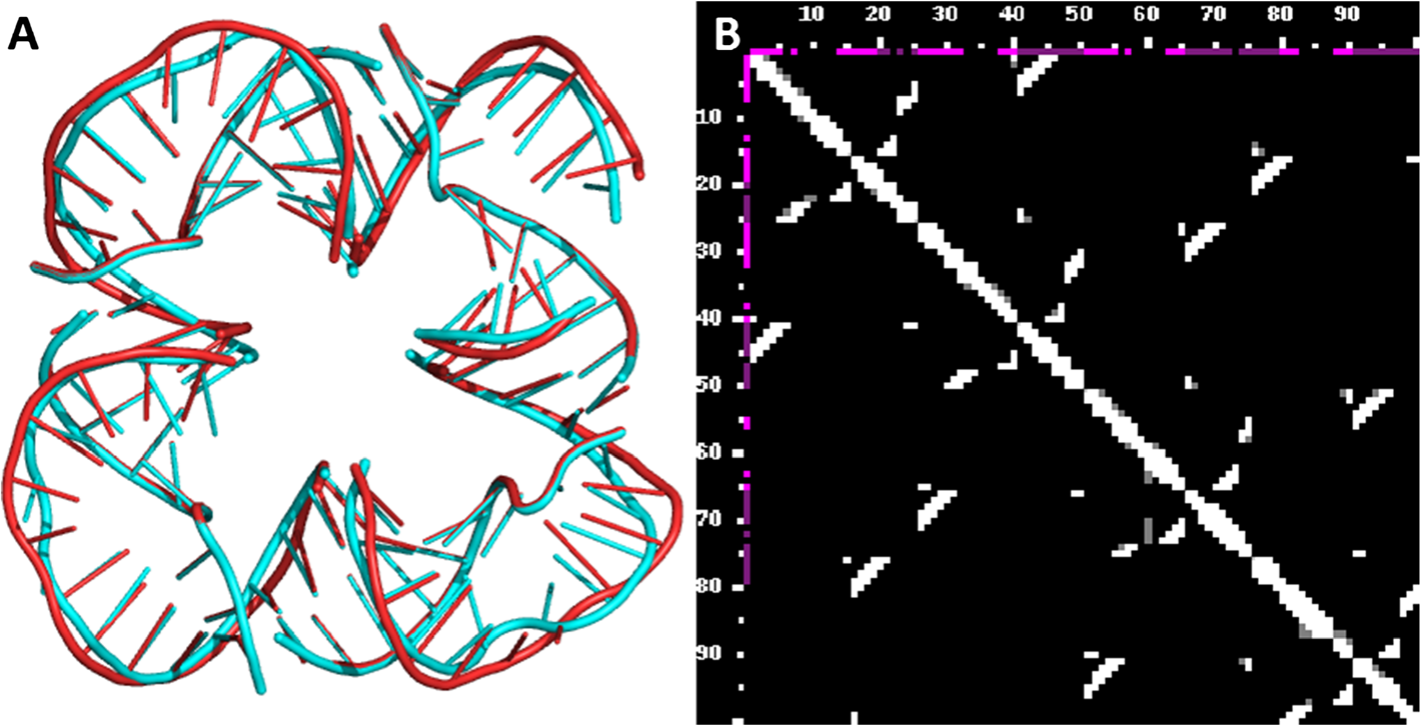
**Self-assembling RNA nano-square versus homology model. A) **The 3D model (red) is compared to the crystal structure (PDB-id: 3P59, [27], cyan). **B) **In the contact map picture, the lower left triangle displays the contacts in the crystal structure, the upper right triangle those in the model. N1/N9 metrics and 9.5 Å threshold was used.

Figure 11 illustrates another example, namely the comparison of one of our models constructed in the framework of the ‘RNA puzzles’ challenge [26]. For this modeling exercise, the secondary structure of the ‘RNA square’ molecule [27] composed of eight individual strands, as well as three-dimensional coordinates of the four strands were provided, and the task was to model the structure and contacts made by the four “missing” strands. Figure 11 (panel A) presents a superpositon of the crystal structure (PDB id 3P59) shown in cyan and a model generated by the Bujnicki group shown in red. This model has been evaluated as the most accurate prediction for this molecule within the RNA Puzzles challenge [26]. The comparison of contact maps with RNAmap2D shows that secondary structures have been modeled correctly, and a significant fraction of tertiary contacts observed in the crystal structure (bottom left) are also present in the theoretical model (top right).

### Comparison of structural ensembles

**Figure 12:**
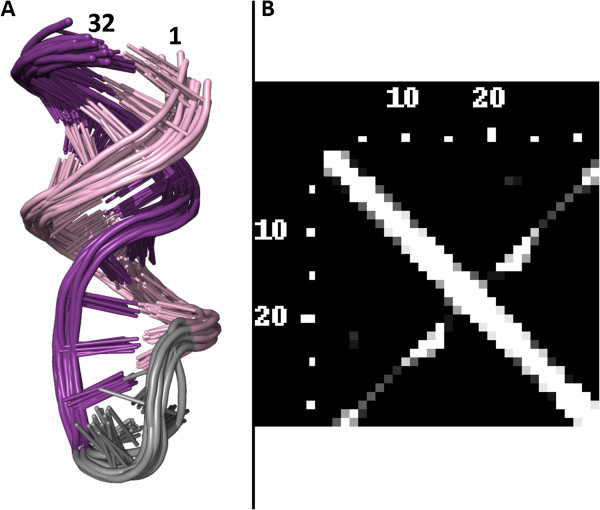
**Statistics of contacts in an NMR file. A) **An AAGU tetraloop hairpin from the protein-RNA complex (PDB code 2LBS_A, for the picture of an entire complex, see Figure 7 – here all NMR models are shown). The RNA molecule is colored according to the secondary structure, in violet and pink. **B) **A statistical contact map of the whole ensemble of RNA chains, 16 models in total. Shades of grey symbolize the percentage of particular contact occurrence in the whole model set. In this case, the C4’ metrics and 12 Å threshold were used to display the variability of contacts in the NMR file analyzed.

Another ability of RNAmap2D is to analyze results of calculations, which typically generate not just one solution, but entire ensembles. Such studies include RNA and RNP structure determination by NMR (review: [28]), and computational structure modeling approaches, such as de novo folding (e.g. with FARNA [29] or iFoldRNA [30] or our in-house method SimRNA [31]) or protein-RNA docking (e.g. using low-resolution method to generate decoys, followed by their scoring and ranking [32]). For such ensembles comprising sets of complexes (from a few to hundreds or even thousands of models) a statistical contact map can be calculated to visualize the frequencies of contacts in the ensemble as shades of grey (Figure 12).

RNAmap2D users can choose between two ways to display contacts that vary through the model ensemble file. In both cases, white fields on the map represent 100% frequency across a model set, and black fields represent no contacts. For contacts that appear only in a fraction of models, one option of visualization presents a relative frequency of contacts, where and shades of grey correspond to intermediate values (the more frequent the contact, the lighter the field). Another option is to present all “partial” contacts by one shade of grey.

In an example analysis, we have analyzed the noncanonical base pairs in a part of the 3^′^ UTR from turnip crinkle virus genomic RNA. This 102-nt structural element binds to the large ribosomal subunit to promote translation. The structure consists of three main helices. In the NMR structure (PDB-ID 2krl), 10 models have been deposited. RNAmap2D can be used to check e.g. whether noncanonical base pairs are maintained in all the models. We used RNAmap2D to generate a contact map for the ensemble and then colored noncanical pairs in blue. We have inspected individual pairs in the map zoomed to a fullscreen mode and generated a statistics map and exported the contact frequencies to an Excel table. In the contact map, regions that vary in the ensemble (e.g. due to increased flexibility) are immediately visible as grey areas. For instance tertiary interactions between hairpin loop 37–43 and the structure (97–101) fluctuate, and base pairing in this region is not stable. In total, we identified 15 noncanonical base pairs, of which four differ in at least one of the 10 models. The three noncanonical pairs G9-G12, G29-U55, and A70-C87 (a sugar-Hoogsteen pair) differ in only one of the models. The fourth pair, cis-Watson-Watson U67-C87, is present in four models only. It varies a lot between the models and the participating bases can also pair in the cis-Watson-Hoogsteen mode. Residues adjacent to A70 and C87 display some flexibility in the contact map as well. This pair is located in an internal loop that is important for switching between translation and replication in the virus [33]. We conclude that RNAmap2D helps to identify regions that undergo conformational changes by highlighting them in the graphical output, and enables their quantitative examination in a tabular report.

## Conclusions

RNAmap2D is a new tool for calculation and visualization of nucleic acid contact and distance maps. Our aim was to facilitate analyses of RNA structures that focus on type and location of short-range interactions, without taking the spatial conformation of the backbone into account. RNAmap2D is also capable of analyzing protein-nucleic acid complexes. RNAmap2D is applicable in various scenarios, ranging from comparison of RNA 2D and 3D structural predictions with each other and with the native structure, to analyses of trajectories from MD simulations of nucleic acid structures, to studies of RNA/DNA-protein and RNA/DNA-ligand interactions and analyses of macromolecular docking experiments.

PROTmap2D and RNAmap2D both provide researchers with an extensive suite of programs for analyses and visualization of macromolecular structures. RNAmap2D runs on any modern operating system, is very fast and has an intuitive interface. Both programs serve as a complete platform that supplements the existing 3D visualization tools, with sophisticated 2D map capabilities.

## Availability and requirements

**Project name**: RNAmap2D

**Project home page**: http://iimcb.genesilico.pl/rnamap2d.html

**Operating systems**: Windows, Linux, MacOSX.

**Programming languages**: Python (main), C/C++ (some parts of external libraries)

**Software packages (Windows, MacOSX)**: None

**Software packages (Linux)**: Python 2.6, Biopython 1.42, PyCogent 1.4, wxPython 2.8.10, PIL 1.1.6, Numeric 24.2, NumPy 1.1.3, PyExcelerator 0.6.3

**Other requirements**: RNAView program is recommended as an optional plugin. Please refer to User’s Manual and README file for installation instructions to be found on the RNAmap2D project home page.

**Hardware requirements**: min. 512 MB RAM, 1GHz CPU or better (2 GB RAM and 2 GHz CPU is recommended).

**License**: RNAmap2D is distributed under free academic license. Please refer to the home page for the license document.

**Restriction for non-academics:** Users willing to use RNAmap2D for non-academic purposes should contact the corresponding author for details. Please note that this license will not affect commercial usage of RNAView. Please contact authors of RNAView for a separate license.

## Abbreviations

PDB: Protein DataBank; PIL: Python Image Library; CASP: critical assessment of techniques for protein structure prediction; CSV: comma-separated values; CT: connectivity table.

## Competing interests

Authors declare that they have no competing interests.

## Authors’ contributions

NS participated in majority of the code implementation, including most of the features specific for RNA. MP co-supervised the work of NS, contributed the code of PROTmap2D, participated in the development of the code, developed the final version of the software, and drafted the manuscript. KR co-supervised the work of NS, and participated in the development of the code. JMB conceived of the study, participated in design and coordination, and edited the manuscript. All authors read and approved the final manuscript.
